# Improved operator agreement and efficiency using the minimum area contour change method for delineation of hyperintense multiple sclerosis lesions on FLAIR MRI

**DOI:** 10.1186/1471-2342-13-29

**Published:** 2013-09-03

**Authors:** David S Wack, Michael G Dwyer, Niels Bergsland, Deepa Ramasamy, Carol Di Perri, Laura Ranza, Sara Hussein, Christopher Magnano, Kevin Seals, Robert Zivadinov

**Affiliations:** 1 Buffalo Neuroimaging Analysis Center, Department of Neurology, School of Medicine and Biomedical Sciences, University at Buffalo, State University of New York at Buffalo, Buffalo, NY, USA; 2 Department of Nuclear Medicine, University at Buffalo, State University of New York at Buffalo, Buffalo, NY, USA; 3 Department of Neuroradiology, IRCCS, C. Mondino, University of Pavia, Pavia, Italy; 4 MR Imaging Clinical Translational Research Center, School of Medicine and Biomedical Sciences, University at Buffalo, State University of New York, Buffalo, NY, USA; 5 School of Medicine and Biomedical Sciences, State University of New York at Buffalo, Buffalo, NY, USA

**Keywords:** Multiple sclerosis, Detection error, Outline error, Rater agreement, Operator agreement, Metric, Jaccard index, Similarity index, Kappa, Lesion, Assessment, Minimum area contour change

## Abstract

**Background:**

Activity of disease in patients with multiple sclerosis (MS) is monitored by detecting and delineating hyper-intense lesions on MRI scans. The Minimum Area Contour Change (MACC) algorithm has been created with two main goals: a) to improve inter-operator agreement on outlining regions of interest (ROIs) and b) to automatically propagate longitudinal ROIs from the baseline scan to a follow-up scan.

**Methods:**

The MACC algorithm first identifies an outer bound for the solution path, forms a high number of iso-contour curves based on equally spaced contour values, and then selects the best contour value to outline the lesion. The MACC software was tested on a set of 17 FLAIR MRI images evaluated by a pair of human experts and a longitudinal dataset of 12 pairs of T2-weighted Fluid Attenuated Inversion Recovery (FLAIR) images that had lesion analysis ROIs drawn by a single expert operator.

**Results:**

In the tests where two human experts evaluated the same MRI images, the MACC program demonstrated that it could markedly reduce inter-operator outline error. In the longitudinal part of the study, the MACC program created ROIs on follow-up scans that were in close agreement to the original expert’s ROIs. Finally, in a post-hoc analysis of 424 follow-up scans 91% of propagated MACC were accepted by an expert and only 9% of the final accepted ROIS had to be created or edited by the expert.

**Conclusion:**

When used with an expert operator's verification of automatically created ROIs, MACC can be used to improve inter- operator agreement and decrease analysis time, which should improve data collected and analyzed in multicenter clinical trials.

## Background

Large agreement differences often exist between operators drawing regions of interest (ROIs) of hyperintense lesions on Fluid Attenuated Inversion Recovery (FLAIR) MRI brain scans of patients with multiple sclerosis (MS) despite training and standards designed to minimize inter-operator variability [1-4]. These differences can be broken down into two categories: a) lesion outline disagreement and b) lesion detection disagreement. Differences in how operators outline a lesion account for the majority of difference (measured by volume) between operators. The size of this outline error (OE) between human experts increases with the level of lesion burden revealed in the patient’s scan. Detection error (DE), the total area of ROIs chosen by one operator that do not match with another operator's ROIs, remains comparatively compensated with increasing lesion burden of the underlying scan. For MRI scans revealing high lesion burden, OE between raters’ ROIs can be much greater than DE [5]. ROIs can be visually very similar, yet have large area differences. As an example, if a 10x10 pixel ROI increased by a half pixel in each direction, the new area would be 21% larger.

Operators often analyze a sequential set of scans that were collected over the course of months or years. Finding a lesion at a given location on a baseline scan is a good indication that a lesion can be found (detected) at the same location on a follow-up scan, though the size and shape (outline) may vary. For this reason, each image must be analyzed and have ROIs drawn for each time point. Despite the similarities between the placements of ROIs between scans of the same individual, there is not a significant time saving in a subsequent lesion analysis compared to the baseline.

A commonly used tool for outlining lesions in MS is the Java Image Manipulation (JIM) software package (version 4.0, Xynapse System, Leichester, UK), which allows an operator to delineate a lesion by clicking near the edge of the lesion. JIM calculates a contour value by choosing the image intensity value associated with the steepest gradient value in a small surrounding neighborhood of the cursor. Using the determined contour value, the program outlines the lesion with an iso-contour curve. This application works well and is an indication that a large percentage of lesion delineations can be represented by a single contour value. There have been many other applications designed to aid operator efficiency and precision in ROI creation such as live wire, active contours, and snakes [6-8]. Lesion growth methods [9,10] have been previously applied to images to delineate MS lesions in order to reduce inter-rater variability. Furthermore, curve evolution has been applied post lesion detection [11]. Re-contouring methods have been applied to auto-propagate an ROI from one phase of a 4D CT image to another [12,13]. The intended application of these tools is to increase operator productivity and reduce inter-operator variability.

Against this background, the Minimum Area Contour Change (MACC) algorithm has been created with two main goals: a) to improve inter-operator agreement on outlining ROIs and b) to automatically propagate longitudinal ROIs from a baseline scan to a follow-up scan (i.e. by “propagate” we mean MACC is run using the baseline ROI set and a follow-up image, to produce ROIs for the follow-up image that have been re-contoured to account for changes in a lesion’s size and shape). For each lesion that has a previously drawn ROI, the MACC software selects a unique/best contour value, and uses the iso-contour curve as the lesion outline. The use of MACC virtually assures an improvement in inter-operator agreement because MACC will settle on a unique ROI from a large range of input ROIs. What is less clear is whether the MACC ROIs are an acceptable replacement for the original ROIs. Therefore, in the present study we test whether MACC meets the following four requirements:

1) Differences between ROIs drawn by MACC and the operators’ original ROIs should be similar to (or smaller than) the differences found between two operators;

2) ROIs outlined by two operators and reanalyzed by MACC should be significantly closer to each other than the comparison between the operators’ original hand-drawn ROIs;

3) If MACC is used to propagate (which includes re-contouring) ROIs to a follow-up image (from the ROIs of the previous scan), differences between ROIs drawn by MACC and the operator’s hand-drawn ROIs (of the current scan) should be similar to differences found between two operators;

4) MACC should process a set of ROIs fast enough to be routinely used by an operator.

## Methods

### MACC algorithm

**Figure 1:**
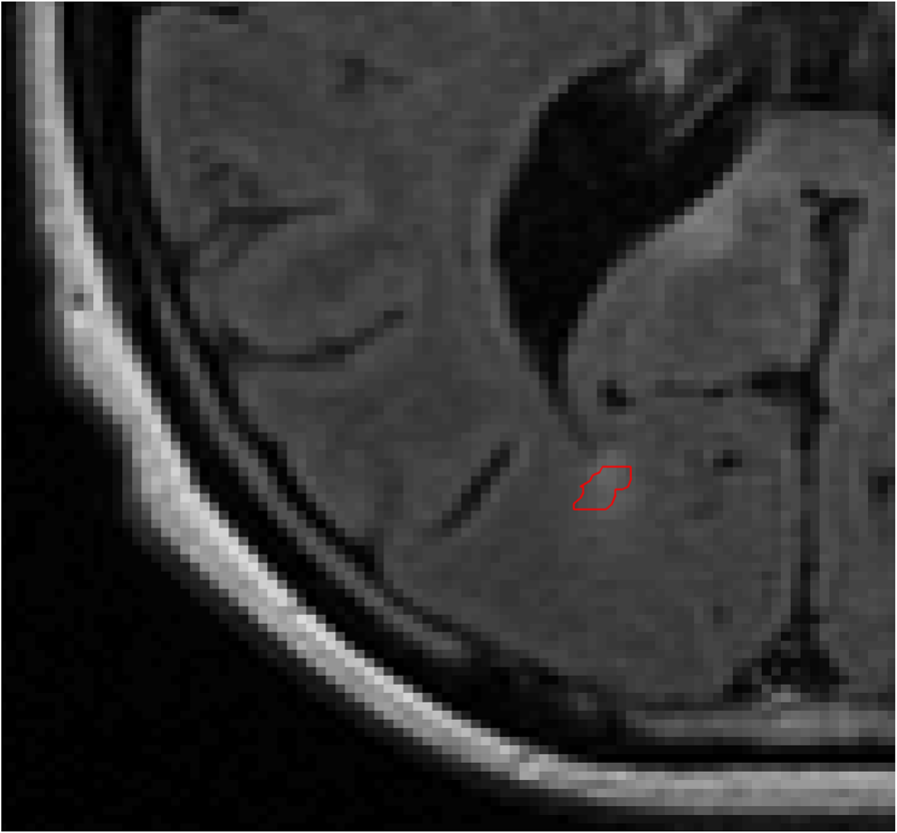
Flair MRI, with a hand-drawn ROI of a lesion shown in lower left.

**Figure 2:**
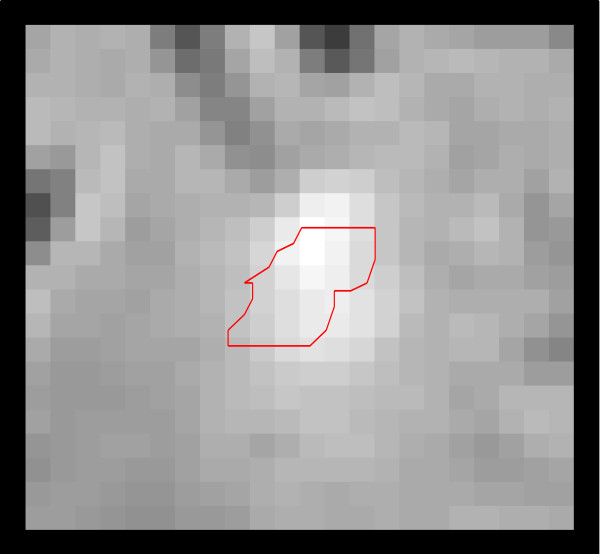
**Close-up view of the hand-drawn ROI from Figure **1**; the entire image displayed represents the rectangular region to bound the MACC ROI.**

**Figure 3:**
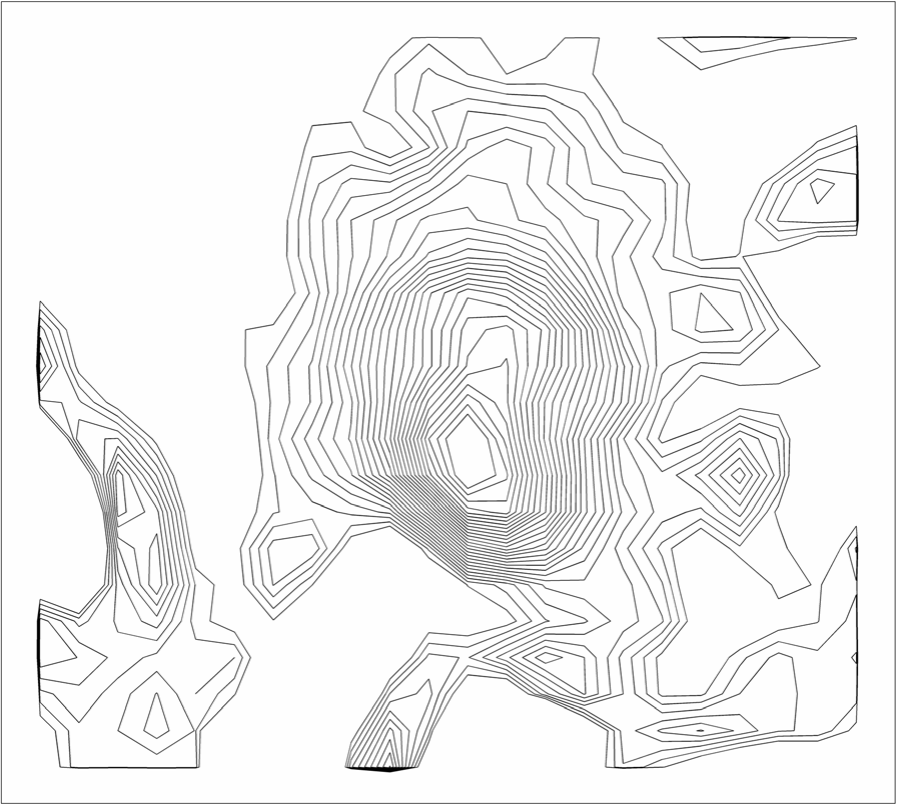
**Iso-contour curves at 50 evenly spaced values.** MACC searches for a pair of neighboring contours that are close together.

**Figure 4:**
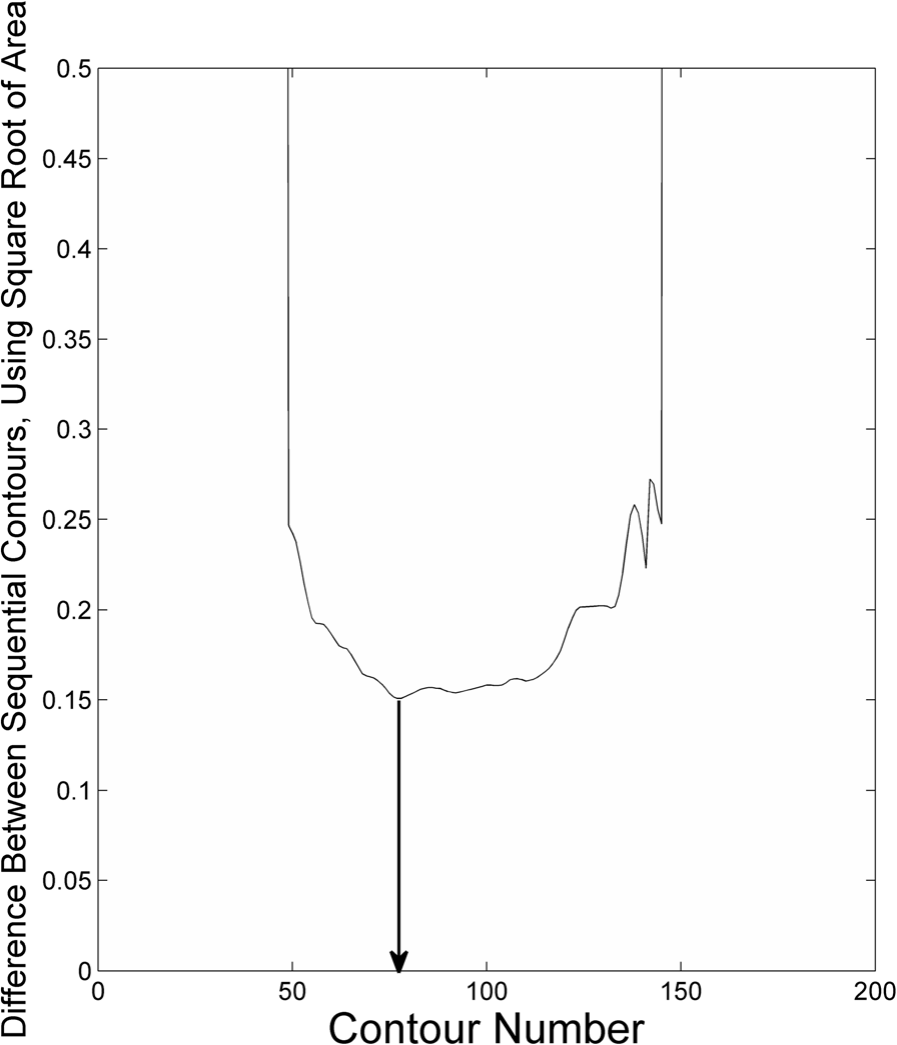
**Graph of contour index number (from low to high) and SqrtArea(L**_**n**_**)-SqrtArea(L**_**n + 1**_**).** The arrow passes through the minimum of the graph, and points to the value of n that is selected by the algorithm.

**Figure 5:**
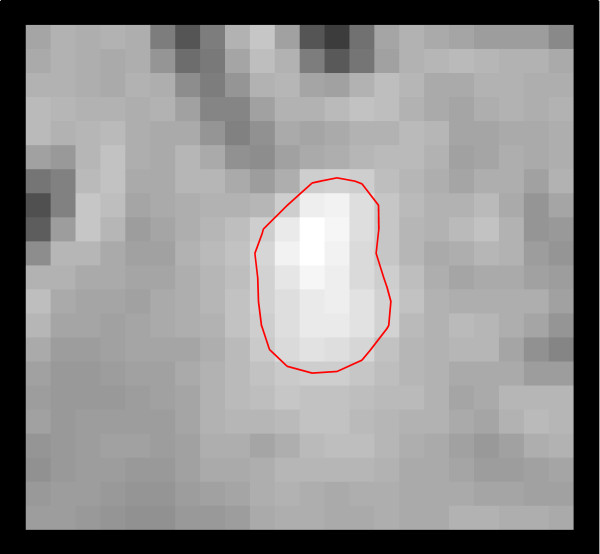
**Close-up view of the MACC ROI for lesion displayed in Figures **1**and **2**.**

The MACC algorithm uses an image and an input set of ROIs to create an output set of ROIs, in the same locations as the input ROIs, but outlined according to the rules of the algorithm. For a given ROI (each individual 2d outline of a single lesion, from a single rater), Figure 1, the algorithm restricts the analysis to a rectangular region around the ROI, Figure 2. Then a large set of iso-contours is created for the restricted image, Figure 3. The program searches for the best contour to be the new outline of the lesion, and applies restrictions to candidate contours. For instance, a contour curve cannot be chosen as the final ROI if it: 1) meets the edge of the bounding box, 2) increases or decreases the ROI size from the original by more than a user specified ratio, or 3) does not intersect with the original ROI. The square root of the total area for all compliant contours at a given contour level, SqrtArea(L_n_), is calculated, where L_n_ represents the nth evenly spaced level value (starting from low to high). The level, L_n_, that minimizes (SqrtArea(L_n_)–SqrtArea(L_n+1_)) is chosen by the program, Figure 4, and the compliant contours at this level are used as the output “MACC” ROI, Figure 5. Using the square root of the contours’ areas was motivated by the desire to find the minimum overall “radial” distance between neighboring ROIs, as would be the case if the set of conforming ROIs were concentric circles. A center could use another metric in place of the radial metric we present provided adequate testing was performed to ensure that it conforms well to how their raters contour lesions. The value should be unique and be efficiently calculated for contours of any shape.

Around a hyper-intense lesion, higher valued contours will be nested within lower valued contours. For a given value, there may be more than one contour, which could be due to high intensity “peaks” found within a lesion. To have multiple ROIs formed from one input ROI is acceptable by the algorithm. Conversely, multiple input ROIs may lead to the same MACC ROI, which would typically fully contain those input ROIs.

Since MACC is dependent on the topography of the image around a lesion, it is possible for it to fail in finding any conforming ROIs. In this case, for purposes of evaluating calculations, the MACC program does not return an ROI. However, our implementation has two other “Failed” ROI modes: one, which returns the original ROI; the other returns a rectangular ROI roughly double the size of the original ROI, which enables quick identification by the operator.

### Algorithm parameters

The area of a MACC ROI was restricted to being greater than 1/3 and less than three times the input ROI. This and selecting how to handle a “Failed” ROI are the only external “tuning” parameters. Remaining parameters are seldom changed and are internal. The MACC algorithm was run with an ROI bounding window that was twice the width and height of the smallest rectangle containing the ROI (with a minimum of 4 mm and maximum of 10 mm padding of the original ROI in any direction). The MACC program selected the best contour curve originating from 250 equally spaced contour values between the median and maximum pixel intensity values within the bounding rectangle.

### Datasets

#### Human operators

To assess the amount by which MACC can improve agreement between operators (requirement 2), a pair of raters (both neurologists) was used who had spent approximately three months in our lab and had over 3 years’ experience in lesion analyses at the time they performed their analyses. No effort was made to optimize inter-rater reliability between the pair of raters, and they should be considered independent experts. The ROIs from the pair of raters were also used in testing whether MACC ROIs were in close agreement with the original raters’ ROIs (requirement 1). To assess whether MACC produces ROIs that are in close agreement with the original raters' ROIs on both the baseline and follow-up scans (requirements 1 and 3), a single rater was used who has several years of experience in our imaging laboratory. All raters were physicians with several years of experience working with MS scans. ROIs were drawn using JIM software and created 2D outlines of lesions on each scan slice.

#### Participants

Two separate datasets were used in testing the algorithm. In Dataset 1, a set of 17 FLAIR MRI images fulfilling the criteria for clinically definite MS were used as in Di Perri et al. [14] and Wack et al. [5], which allowed comparison between a pair of human operators’ ROIs with and without the use of MACC (requirements 1 and 2). In Dataset 2, a set of 12 baseline and matching 12 month follow-up FLAIR MRI scans of relapsing-remitting MS patients were used to assess the agreement between MACC ROIs and the original raters' ROIs (requirements 1 and 3). To avoid any potential bias in data analysis, we placed images in a halfway space during co-registration. Briefly, we used FSL’s FLIRT linear registration tool (http://www.fmrib.ox.ac.uk/) [15,16] to estimate a forward transform from baseline to follow-up, and a backward transform from follow-up to baseline. We then took the halfway transforms for each, and used these to re-sample their respective images into a common space. Participants gave informed consent and study protocols were approved by the University at Buffalo’s Health Sciences Institutional Review Board.

#### Scanning protocol

All scans were acquired on a GE 3 T Signa Excite HD 12.0 Twin Speed 8-channel scanner (General Electric, GE, Milwaukee, WI). The scanner has a maximum slew rate of 150 T/m/s and maximum gradient amplitude of 50 mT/m in each orthogonal plane. A multi-channel Head and Neck (HDNV) coil was used for all sequence acquisitions. Scan voxel dimensions were .86 × .86 × 2.92 mm and .94 × .94 × 3.0 mm for the first and second sets of scans, respectively. Scans were collected in a clinical setting with a standardized protocol including double-echo proton density (PDw) and T2-weighted (T2w) spin echo (SE), a FLAIR, and T1-weighted (T1w) SE pre- and post-contrast injection, all prescribed axial with subcallosal alignment. Acquisition parameters were defined as follows: for the PD-T2 repetition time (TR) = 3000 ms, first/second Echo time (TE) = 12/95 ms, FA = 90°, echo train length (ETL) = 14, Bandwidth (BW) = 20.83 Hz/pix, field of view (FOV) = 240 mm, acquisition matrix = 256 × 256, phase field of view (pFOV) = 0.75 for an in plane resolution of 0.94 × 0.94 mm, slice thickness = 3 mm for 46 slices, NEX = 1 for an acquisition time of 4:31 min; for the FLAIR TR = 8500 ms, TE = 120 ms, FA = 90°, BW = 27.78 Hz/pix, FOV = 240 mm for the first dataset and FOV = 220 mm for the second one, acquisition matrix = 256 × 256, pFOV = 0.75 for an in plane resolution of respectively 0.94 × 0.94 mm and 0.83 × 0.83, slice thickness respectively of 3 mm and 2.92, for 46 slices, NEX = 1 for an acquisition time of 4:16 min; for the T1w SE pre- and post-contrast injection TR = 550 ms, TE = 12 ms, FA = 60°, FOV = 240 mm, acquisition matrix = 256 × 256, pFOV = 0.75 for an in plane resolution of 0.94 × 0.94 mm, slice thickness = 3 mm for 46 slices, NEX = 1 for an acquisition time of 3:47 min.

### Assessment measures

In addition to the Similarity Index (SI) we use the measures of Detection Error (DE) and Outline Error Rate (OER) [5], to compare ROIs. We define a connected region as a single region (blob) contained in the union of raters’ ROIs. We label each connected region, as CR1, CR2, or CR12 based on whether it is composed of ROIs solely from rater 1, rater 2, or from both raters 1 and 2, respectively. For a given connected region, *cr*, we denote the ROIs created by rater 1 contained within *cr* as R1(cr). Similarly, R2(*cr*) represents the ROIs created by rater 2 within *cr*, and hence *R*1(*cr*) ∪ *R*2(*cr*) = *cr*. DE is the total area of ROIs of lesions that were marked by either, but only one, of the raters, and is sensitive to whether raters mark the same hyper-intense regions as a lesion:

$$\mathrm{DE}=\sum_{\text{cr}\;∊\;\text{CR}1\;\text{or}\;\text{CR}2}\left|\mathrm{cr}\right|,$$

where |*cr*| represents the area of the connected region, *cr*; and *cr* ∊ *CR*1 *or CR*2 represents the set of connected regions that can be labeled as either CR1 or CR2.

OE is the difference in area between the union and intersection of ROIs of lesions that were marked by both raters, and is sensitive to how well two raters agree in determining and marking the edge of a lesion:

$$\mathrm{OE}=\sum_{\mathrm{cr}\in \mathrm{CR}12}\left(\left|\mathrm{cr}|-|R1\left(\mathrm{cr}\right)\cap R2\left(\mathrm{cr}\right)\right|\right)$$

where |R1(*cr*) ∩ R2(*cr*)| represent the area of the intersection of rater 1 and rater 2’s ROIs within *cr*, respectively.

We calculate the mean total area (MTA) of the two raters ROIs as: MTA = (1/2)(|R1| + |R2|), where |R1| and |R2| represent the total area of rater 1 and rater 2’s ROIs, respectively.

Similarity Index (SI) is commonly defined as 2 times the area of the intersection of the raters’ ROIs, divided by sum of the area of the raters ROIs [17]. Defining $\mathrm{OER}=\frac{\mathrm{OE}}{\mathrm{MTA}}$, SI is related to DE, OER, and MTA as (see appendix):

$$\mathrm{SI}\left(R1,R2\right)=1-\left(\frac{1}{2}\right)\mathrm{OER}‒\left(\frac{1}{2}\right)\frac{\mathrm{DE}}{\mathrm{MTA}}$$

We expect OER to be very sensitive to the changes in ROIs in the presented experiments because MACC attempts to improve agreement between ROI sets through the re-contouring (outlining) of ROIs. We do not expect much change in DE, since MACC attempts to create an ROI at each location given by the input ROI. Hence, given SI’s dependence on both DE and OER, we expect SI to be less sensitive than OER to measuring the changes in ROIs made by MACC.

We will also plot the histogram of $\frac{\left(\left|R2\left(\mathrm{cr}\right)|-|R1\left(\mathrm{cr}\right)\right|\right)}{\left|\mathrm{cr}\right|}$ for all connected regions that can be labeled as CR12 regions, which we term the Outline Error Distribution (OED) graph. If MACC draws the majority of ROIs as either larger or smaller than the original set of ROIs, the OED graph will be biased to one side of 0. If operators draw most ROIs identically, there will be a large peak at 0.

### Software assessment

We use r_1_ and r_2_ to denote the two raters used on Dataset 1, and r to denote the single rater used on Dataset 2. We use im to denote an image from Dataset 1 (single time point, with 17 participants), and im_b_, and im_f_ to denote the baseline and follow-up images from Dataset 2. The ROIs created by rater r on the baseline image (im_b_) from Dataset 2 (with 12 participants) are denoted as ROI(r, im_b_). ROIs created by MACC on the follow-up image (im_f_) are denoted as MACC(ROI(r, im_b_), im_f_). Other combinations will follow this same convention. We evaluate our software based on four assessments that correspond to our four requirements.

1) MACC ROIs are acceptable replacements for the original ROIs:

Requirement: Differences between ROIs drawn by MACC and the operators’ original ROIs should be similar to (or smaller than) the differences found between two operators.

Assessment: Measure the agreement using DE, OER, and SI for all three raters between their original ROI and the MACC ROI created on the same image. That is, using Dataset 1 compare ROI(r_1_, im) vs. MACC(ROI(r_1_, im), im), and ROI(r_2_, im) vs. MACC(ROI(r_2_, im), im). With Dataset 2 compare the agreement between: ROI(r, im_b_) vs. MACC(ROI(r, im_b_), im_b_). All comparisons using MACC will then be compared with the agreement seen between two expert raters without MACC: ROI(r_1_, im) vs. ROI(r_2_, im).We will show the OED graph for the comparisons and inspect for signs of asymmetry or bias.

2) MACC improves inter-rater agreement:

Requirement: ROIs outlined by two operators and reanalyzed by MACC should be significantly closer to each other than the comparison between the operator’s original hand-drawn ROIs.

Assessment: Using Dataset 1, measure the agreement using DE, OER, and SI between MACC(ROI(r_1_, im), im) and MACC(ROI(r_2_, im), im), and compare with the agreement seen between the two expert raters without MACC: ROI(r_1_, im) vs ROI(r_2_, im). Create, inspect, and compare the outline error distribution graph for both agreement comparisons.

3) MACC can propagate ROIs to follow-up images:

Requirement: If MACC is used to create ROIs for a follow-up image (from the ROIs of the previous scan), differences between ROIs re-contoured by MACC (on the follow-up image) and the operator’s hand-drawn ROIs (on the follow-up image) should be similar to differences found between the two operators.

Assessment: Using Dataset 2, measure the agreement using DE, OER, and SI between ROI(r, im_f_) and MACC(ROI(r, im_b)_, im_f_). Additionally, measure the agreement between MACC(ROI(r, im_f_), im_f_) and MACC(ROI(r, im_b_), im_f_).

4) MACC executes quickly:

Requirement: MACC should process a set of ROIs fast enough to be routinely used by an operator.

Assessment: Measure the mean time for MACC to process the ROIs in assessment 3.

### Post-hoc analysis

We examined the ROI files from 424 follow-up FLAIR scans of patients with MS, where the original follow-up ROI file was created by MACC, and subsequently edited by an expert. We then determined the percentage of ROIs that MACC produced that were found acceptable without adjustment, and the percentage of the final number of ROIs that were either edited or created by the expert.

## Results

### Assessment 1: MACC ROIs are acceptable replacements for the original ROIs

**Figure 6:**
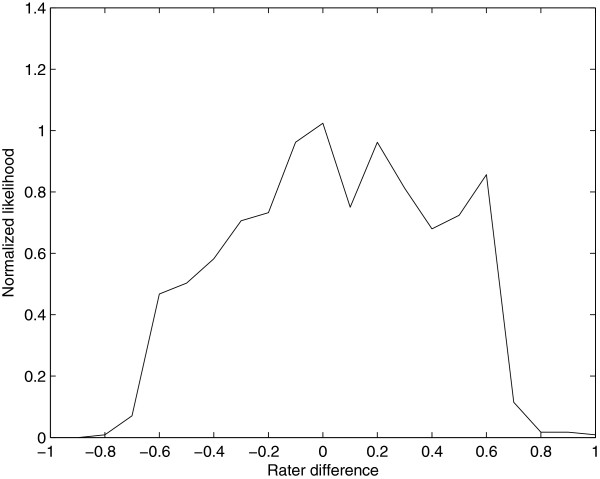
**The Outline Error Distribution graph comparing ROI(r, im**_**b**_**) and MACC (ROI(r, im**_**b**_**), im**_**b**_**).** Negative abscissa values indicate the original ROIs are larger than the MACC ROIs, and positive values indicate the opposite.

For Dataset 1, 1710 ROIs were drawn by either rater from 17 scans with mean LV of 15,084 mm^3^. Values for DE, OER, and SI for the two raters’ original ROIs and the corresponding MACC ROIs from Dataset 1 (Rows 2 and 3 of Table 1) were better than those found between two raters without MACC (Row 1, Table 1). Mean lesion volumes (LVs) measured from the rater and MACC processed ROIs were 8443 mm^3^ and 7817 mm^3^, respectively, for Dataset 2. ROIs from this dataset using the single rater were also processed with MACC and compared—using DE, OER, and SI—to the originals, see Row 1, Table 2. In total 1135 2D ROIs were evaluated from 12 scans. The program failed in creating a MACC contour for two ROIs that were both smaller than 3.0 mm^2^ in size. The average DE, OER, and SI values between the original and MACC ROIs were in each case better than the corresponding values found in the comparison between the two raters (Row 1, Table 1). The distribution of sizes between ROIs drawn by the operator and the MACC program were evenly distributed as shown by the OED graph, Figure 6; coefficient of variation (COV) of MTA between the original and MACC ROIs was COV = .10. The Pearson correlation of the individual ROI areas of the original and MACC programs = .99 ( p < < .0001 ).

**Table 1:** Cross-sectional Data, Dataset 1

**ROI sets**	**Detection error (mm**^**2**^**)**	**Outline error rate**	**Similarity index**
ROI(r_1_, im) v. ROI(r_2_, im)	747	.41	.64
ROI(r_1_, im) v. MACC(ROI(r_1_, im), im)	1	.40	.80
ROI(r_2_, im) v. MACC(ROI(r_2_, im), im)	1	.39	.80
MACC(ROI(r_1_, im), im) v. MACC( ROI(r_2_, im), im)	714	.21	.72

**Table 2:** Longitudinal Data, Dataset 2

**ROI sets**	**Detection error (mm**^**2**^**)**	**Outline error rate**	**Similarity index**
MACC(ROI(r, im_b_), im_b_) v. ROI(r, im_b_)	~0 (.24)	.37	.82
MACC(ROI(r, im_b_), im_f_) v. ROI(r, im_f_)	473	.41	.63
MACC(ROI(r, im_b_), im_f_) v. MACC(ROI(r, im_f_), im_f_)	450	.18	.74

### Assessment 2: MACC improves inter-rater agreement

Using Dataset 1, ROIs from two raters were processed through MACC together with the original image (same for both raters), on which the ROIs were created. 579 ROIs had a detection error with the other operator. The median ROI size having a detection error was 12 mm^2^. DE, OER, and SI for both pre- and post-MACC processing are reported in Table 1. While DE remained roughly the same, both OER and SI, measured between the two raters ROIs (Row 4 vs. Row1, Table 1), improved for *all* 17 ROI sets after MACC processing. OER was approximately cut in half, with min/mean/max decreases of .05/.19/.35, (p < .0001, paired t-test). SI increased on average 12.5%, with min/mean/max increases of .01/.08/.16, (p < .0001, paired t-test).

**Figure 7:**
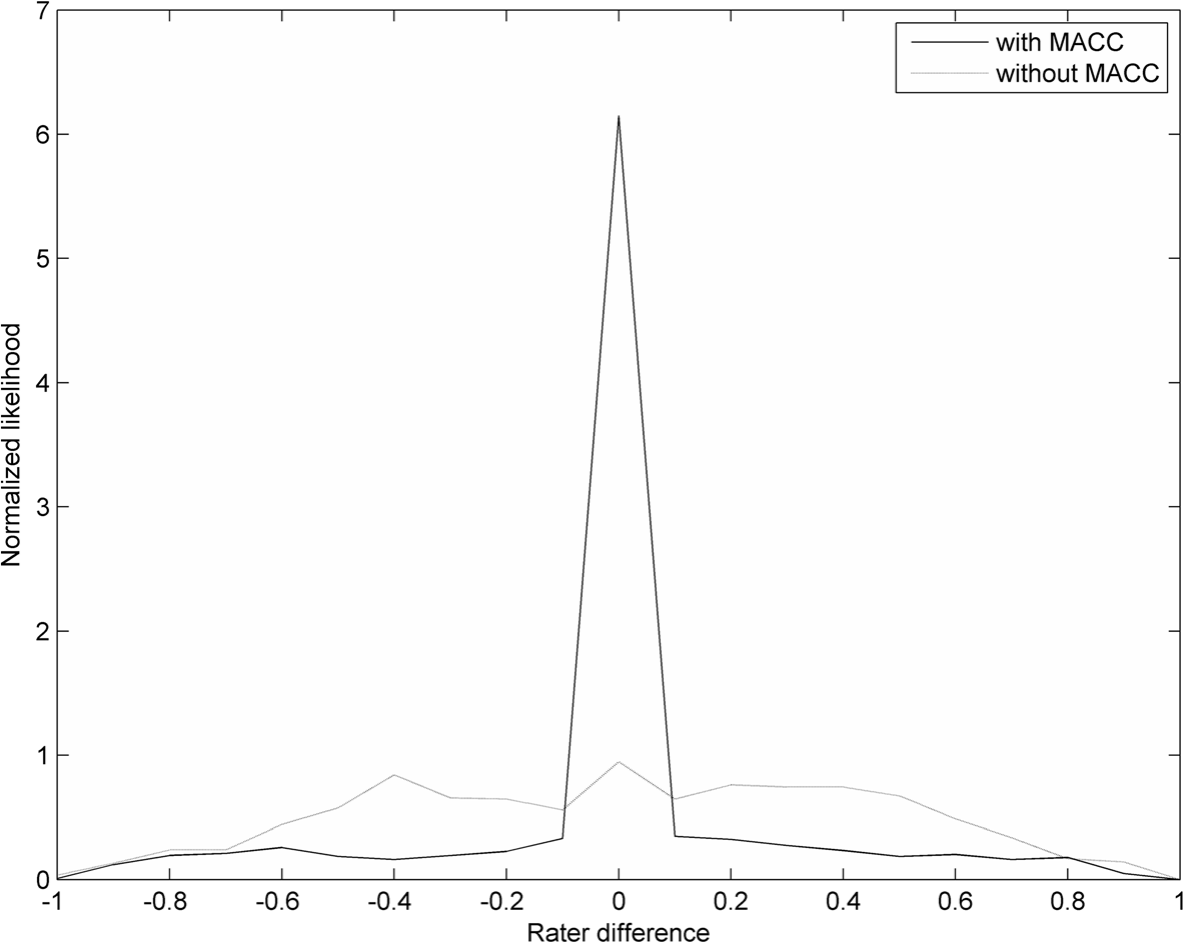
**The graph with the sharp peak is the Outline Error Distribution (OED) graph of MACC (ROI(r**_**1**_**, im), im) and MACC (ROI(r**_**2**_**, im), im), and indicates that most compared ROIs have near zero area disagreement.** The flatter graph is the Outline Error OED graph of ROI(r_1_, im) and ROI(r_2_, im), and indicates a broad range of area disagreement between corresponding ROIs. Negative abscissa values indicate that operator 1’s ROIs are larger than operator 2’s ROIs, and positive values indicate the opposite.

The OED graph between the operators’ original ROIs and the MACC ROIs is shown in Figure 7. MACC has over six times the number of ROIs between raters with near zero differences, and has fewer ROIs with size mismatches except for extreme ROI size differences, where MACC and the original ROIs are roughly evenly distributed.

### Assessment 3: MACC can propagate ROIs to follow-up images

**Figure 8:**
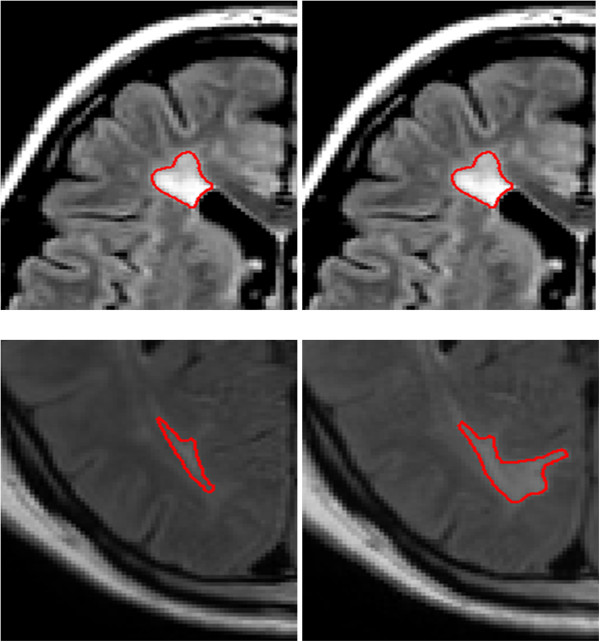
**The left images are from a FLAIR MRI baseline scan of a patient with MS, and the right images are the matching planes at 3 month follow-up.** MACC was used to propagate ROIs drawn of the lesions on the baseline scan to automatically create the ROIs on the follow-up scan. Since MACC creates the lesion ROIs based on the underlying image topography (in this case the follow-up scan), the automatically drawn follow-up ROIs accurately reflect both the changes in the size and shape of the lesion.

An example of MACC used for lesion propagation is given in Figure 8. Mean LV as measured by the unprocessed raters’ ROIs for the follow-up scans in Dataset 2 was 5822 mm^3^. Mean values for DE, OER and SI between MACC(ROI(r, im_b_), im_f_) and ROI(r, im_f_) are also given in Table 2. The average OER was .41 (Row 2, Table 2) and was the same as between two raters (Row 1, Table 1). Average DE was better (37% lower) than between two raters, however, SI was similar, .63 vs. .64.

When MACC ROIs originating from the baseline image but processed with the follow-up image were compared to MACC processed ROIs created from the rater’s original ROIs from follow-up image, SI improved for *all* 12 comparisons (Row 3, Table 2), compared to using the original rater ROIs from the follow-up scan (p < .0001, paired t-test). That is SI was better between MACC(ROI(r, im_b_), im_f_) and MACC(ROI(r, im_f_), im_f_ ) than between MACC(ROI(r, im_b_), im_f_) and ROI(r, im_f_). The min/mean/max improvement of SI were 05/.11/.26, respectively. Likewise, OER improved for *all* scans with min/mean/max decreases of .08/.23/.41 (p < .0001, paired t-test).

### Assessment 4: MACC executes quickly

Average running time for the MACC software on the 34 trials performed in Assessment 3 was 23.5 seconds. For this calculation the program did not make use of parallelization, and was performed on a Dell (Round Rock, TX) R610 server.

### Assessment post-hoc: MACC propagated ROIs are predominately acceptable

For an illustration of MACCs real-world utility, an analysis was performed of ROIs drawn on 424 follow-up FLAIR MRI scans of patients with MS where MACC was used to create initial sets of ROIs that were subsequently edited so every ROI was compliant with our center’s standards. In total, the MACC program generated 56,656 ROIs, of which 91% needed no modification by the trained operator to meet our center’s standards. The final edited set consisted of 56,615 ROIs, of which only 9% were drawn by the operator. The plan for this analysis was made after the final ROI sets were created, and hence the operator was not biased to accept MACC ROIs, nor was the operator acting under time constraints.

## Discussion

Incorporating MACC in the analysis of MS lesions not only achieves the more important goal of improving inter-operator agreement; but can also provide a significant time savings for the neuroimager. The above assessments of MACC established that: 1) MACC-created ROI’s can be used in place of ROIs drawn by an expert operator; 2) inter-operator agreement between MACC-created ROIs is improved compared to the original ROIs; 3) significant time savings can be achieved for the lesion analysis of follow up scans; 4) MACC can be routinely used by any MS MRI reading center since the computational burden is low. Our post-hoc analysis on 424 follow-up scans showed that the vast majority of MACC propagated ROIs (91%) need no further expert adjustment.

### MACC ROIs are acceptable replacements for the original ROIs

ROIs that have been created by MACC are in close agreement with ROIs that were used as input. The comparison measures between the rater-drawn ROIs and the output MACC ROIs, when the rater ROIs are used as input, were better for all three raters (Rows 2 and 3, Table 1; Row 1, Table 2), than between the two expert raters’ original ROIs (Row 1, Table 1). DE in these comparisons is the total size of the rater-drawn ROIs for which MACC failed to produce an ROI, and is near zero. Given the dependence SI has on OER and DE, SI = 1-1/2*OER–½* DE/MTA, the high values for SI are largely attributable to the low DE.

A crucial point is that for MACC ROIs to be useful as a replacement for the original ROIs, the differences in ROIs (un-processed and MACC-processed ROIs) should be less than the expected difference between (un-processed) ROIs drawn by two experts. This is confirmed by OER being slightly higher (worse) for the two expert raters. Furthermore, the OED graph between the rater-drawn ROIs and the MACC ROIs is roughly symmetric and does not reveal an obvious bias (Figure 6). This demonstrates that MACC does not favor the creation of ROIs that are in general either larger or smaller than the expert rater’s ROIs used as input. We conclude that MACC ROIs are an acceptable replacement for the operators’ ROIs.

### MACC improves inter-operator agreement

The use of MACC improves inter-operator agreement when applied to the ROIs from two different raters on the same scans (Assessment 2). MACC in this assessment reduced average OER from .41 to .21 (p < .0001, paired t-test), resulting in the average SI value increasing from .64 to .72, (p < .0001, paired t-test). MACC improved operator agreement for all 17 ROI sets in the assessment. If raters already had perfect agreement, then MACC obviously would not be able to provide any improvement. However, we note that the closer the ROIs of two raters are to each other, the higher the likelihood that MACC will settle on the identical ROI (note the spike of 0 disagreements in Figure 7 after using MACC). Therefore, we consider it a strength of our study that we did not make an attempt to calibrate the raters prior to the study, and is an indication that MACC could likely be utilized effectively in multi-center studies to reduce rater variability.

### MACC can propagate ROIs to follow-up images

Rater-drawn ROIs on the follow-up image are in close agreement with MACC ROIs created from the baseline ROIs together with the follow up image as input, MACC(ROI(r, im_b_), im_f_). That is, the baseline ROIs are re-contoured to account for the changes in the size and shape of the lesions seen on the follow-up image. DE was roughly half that observed between the two independent raters, while OER was the same (.41). The lower value for DE reflects the advantage a single rater has in agreeing on the identification of lesions on baseline and follow-up scans compared to two independent raters identifying the same region as a lesion on the same scan. We believe the most relevant value for this comparison is OER, which indicated an equal performance.

Significantly closer agreement is seen between MACC(ROI(r, im_b_), im_f_) and MACC(ROI(r, im_f_), im_f_) rather than ROI(r, im_f_). The OER value for this new comparison (Row 3, Table 2) is lower (.18) and results in the higher SI value of .74 because DE remains essentially unchanged. To put the low value of OER = .18 into perspective, if DE equaled zero then SI would equal .91.

That DE is lower (better) for the follow-up image using one-rater than between the two-raters on a single image is not a relevant comparison. DE for the follow-up image mainly represents the area of new or disappearing lesions and is driven by pathology, whereas DE between the two raters represents their disagreement on which regions to mark as lesion. The important point about DE for the longitudinal study is that after editing the follow-up image, DE should be near zero. In fact, DE can be made to equal 0 by only adding or deleting ROIs (i.e., without having to change the shapes of any MACC propagated ROIs). To have DE equal zero after editing, is only to say that the same rater would choose all of the same regions to mark as lesion whether starting from propagated MACC ROIs, or starting from scratch. In practice, of course, operators should add, delete, and modify ROIs until they are 100% satisfied. Our argument above is to indicate that propagated MACC ROIs provide a good starting point for doing so. MACC was designed to be a simple and effective tool that improves rater agreement through the improvement of OE and the propagation of lesions. MACC does not delineate new lesions, nor does it automatically remove the ROIs of those that have disappeared. This aspect is left entirely to the expert human rater, or could be addressed with other software designed to improve DE.

### MACC executes quickly

The improved inter-rater reproducibility should be worth MACC’s short execution time, which averaged less than 24 seconds, and time needed to inspect the final ROIs. Our post-hoc analysis on a high number of scans indicates that the vast majorities of MACC ROIs are of use to an expert, and only a small percentage of ROIs needed to be created or edited for the final analysis, hence providing a time savings.

### Choice of MACC parameters

An advantage of MACC is that it has few “tuning” parameters, and in fact only two settings are typically used, and *these will only affect a small percentage of ROIs*. The first parameter *only bounds* the size of an output ROI based on the size of the input ROI. In the presented assessments, MACC ROIs were constrained to be at least 1/3 and less than three times the input ROI. These values were chosen prior to testing as reasonable bounds that would handle the vast majority of lesions. The second parameter determines the output ROI if MACC does not find a solution otherwise. In these instances, MACC is designed to “fail” in one of three modes by returning: no ROI, the original ROI, or an easily identifiable rectangular ROI. This study chose to have MACC “fail” by producing no ROI, to enable some study calculations. The major precondition for MACC to be used to propagate ROIs between longitudinal scans is that the scans must be aligned with each other.

For an analysis center to improve inter-operator reproducibility, which is a concern in large studies requiring multiple operators, we recommend using MACC as a final pass after operators have created their ROIs. In this case, we suggest that MACC be run so the area of the output ROI is at least within the bounds of 1/2 and two times the original; if MACC fails to find a solution, the original ROI should be returned. The MACC ROIs should be inspected by the operator to ensure conformance with the center’s standards, but used in this way very few ROIs should require any modifications. If MACC is used to propagate ROIs to a follow-up scan we recommend that MACC in this case be run in the failure mode that produces rectangular ROIs to mark locations where the software was unable to find a proper MACC ROI. Afterwards, an operator can quickly identify, examine, and fix the rectangular ROI. Locations where a lesion disappeared will often be locations where MACC is unable to produce a conforming ROI and will be marked with a rectangle.

An imaging center could use another metric in place of the radial metric we presented, provided adequate testing was performed to ensure that it conforms well to how their raters contour lesions. We choose this “radial” metric for its simplicity. The contour curves are evenly spaced based on intensity values, but are physically closer on the image as the image gradient increases. Varying the parameters for MACC will have only small effects, which we believe is desirable. The main purpose in *bounding* the extent to which a lesion can grow or shrink is to provide an easy way to avoid consideration of other strong image gradients such as the outline of the brain, or a small single spot of the lesion that has very high intensity. We did not try to optimize parameters such as the parameter limiting the extent to which the lesion can grow or shrink. That is, the results that we present represent what one could reasonably expect by implementing the MACC method themselves; the results are not from specialized modification of tuning parameters for our particular set of data. An investigator implementing the MACC method may want to test other bounds, perhaps including additional minimum or maximum sizes, etc.

### Comparison automated lesion segmentation algorithms

We classify MACC as a lesion “propagation” tool, *not* an automated lesion segmentation algorithms (ALSAs) [18], and in this capacity it is only useful for determining ROIs for follow-up images in a longitudinal study. In contrast ALSAs are usually but not always tested on single time point images [19]. ALSAs, in essence, attempt to solve a harder problem: detect and outline lesions, without knowledge of where previous lesions were marked for that patient. But, if one considers a longitudinal study with four time points, then on ¾ of the scans traditional ALSAs are not using the very valuable information of how previous scans were marked. Region growing (RG) and fuzzy connectivity (FC) methods [20-22] have perhaps the closest similarity to MACC software, in that they can start with a selection of lesion and non-lesion voxels. The resulting segmentation of a lesion using FC or RG will be the collection of voxels that best optimizes the algorithms selection criteria. As with ALSAs methods, these have been evaluated in terms of SI values or calculation of total lesion volume, on datasets from the individual researcher’s labs. This makes comparison between methods difficult. One difficulty is that agreement in terms of SI is better on high lesion load images than on low lesion load images [5,23-25]. This is a benefit in breaking SI into OER and DE as SI = 1-.5 OER-.5 (DE/MTA), noting that MTA is an assessment of lesion load. Many ALSA methods have reported similar performance in terms of SI than we observed between our two raters [23-25]. Note that, since MACC provides contours that delineate lesions with sub-voxel accuracy, SI, OER, and DE for MACC are calculated at a sub-voxel level. In contrast, ALSAs typically work at a voxel level. For lesions with a high contrast between edge and background tissue, MACC, FC, and RG methods should have similar results. We view MACC as complementary to ALSAs, and speculate it could be applied as a post-processing step to give better agreement with a human rater whose ROIs were also processed by MACC.

Finally in our post-hoc assessment performed on 424 follow-up FLAIR images and over 55,000 ROIs, MACC was able to produce 91% of the required ROIs such that they did not need further editing to meet our centers stringent standards. Having to only draw/edit only ~9% of the ROIs represents a significant time savings for an analysis center.

### Assessment indices

We included SI as a performance index for MACC because SI is commonly used for comparing image masks. We prefer DE and OER values because they are descriptive of how MACC improves agreement and are more resistant to the influence of the underlying ROI set’s MTA. MACC was designed to improve OER and not DE. SI reflects the proportional size difference between ROI sets. That MACC performs well as measured by SI reflects the overall dominance of OER over DE in the calculation of SI, especially for ROI sets with high MTA. Despite the low impact DE may have on SI, DE is an important measure. For instance, DE will be much more sensitive than SI for determining whether raters agree in marking the same small lesions, which is important if an outcome for an analysis is the number of “new” lesions.

### Uses of MACC

We have demonstrated using MACC to both improve inter-operator agreement in creating ROIs, and for efficiency when ROIs on follow-up scans are required. Furthermore, the concept of MACC can be readily used in other medical imaging scenarios. MACC was shown to improve measurement reliability and reproducibility of cerebral spinal fluid flow [26]. MACC could potentially be used for evaluation of “blackholes” [27], however because of the greatly reduced number of contours per image, the time savings would not be significant.

## Conclusion

We have introduced a method that is both well-defined and easy to implement, which drastically reduces the error associated with multiple operators performing lesion analysis. The method also provides significant time savings for operators creating ROIs on follow up scans, assuming the spatial registration of MR images. The method is fast and enables better precision in subsequent steps of lesion activity analysis.

## Appendix

Letting I be the intersection of both raters’ ROIs, U be the union of the rater ROIs, SI can be expressed as SI = 2I / (U + I). By adding and subtracting U to the numerator, SI = ((U + I)-(U-I)) / U + I. Since DE + OE = U-I (i.e., total error equals the total difference between ROIs), MTA = ½( U + I), and OER = OE/MTA:

$$\begin{array}{ll} \mathrm{SI}\left(R1,R2\right) & =1-\frac{U‒I}{U+I}=1-\frac{\mathrm{OE}+\mathrm{DE}}{2\mathrm{MTA}}\\ & =1-\left(\frac{1}{2}\right)\mathrm{OER}‒\left(\frac{1}{2}\right)\frac{\mathrm{DE}}{\mathrm{MTA}} \end{array}$$

We represent OE/MTA as a single variable (OER) since we expect OE to increase as MTA increases. That is, the total outline error area (OE) of two sets of ROIs increases with the number and sizes of ROIs in the sets. We do not necessarily believe that raters will misidentify a region as being a lesion is strongly dependent on the lesion load, hence the contribution to SI from DE is - .5 (DE/MTA). For this reason, we expect higher SI between raters of algorithms evaluated on higher lesion load images, as is consistently observed.

## Competing interests

The authors declared that they have no competing interests.

## Authors’ contributions

Algorithm development: DSW; study design: DSW, MGD, NB, SH, RZ; image analysis: DR, CDP, LR, SH; image processing: DSW, MGB, NB, CM, KS; initial manuscript draft: DSW; manuscript review and revision: All authors read and approved the final manuscript.

## Pre-publication history

The pre-publication history for this paper can be accessed here:

http://www.biomedcentral.com/1471-2342/13/29/prepub

## References

[B1] Achiron A Brain MRI lesion load quantification in multiple sclerosis: a comparison between automated multispectral and semi-automated thresholding computer-assisted techniques Magnetic resonance imaging 2002 20 10 713 720 10.1016/S0730-725X(02)00606-9 12591567

[B2] Filippi M Effect of training and different measurement strategies on the reproducibility of brain MRI lesion load measurements in multiple sclerosis Neurology 1998 50 1 238 244 10.1212/WNL.50.1.238 9443486

[B3] Jackson EF Accuracy and reproducibility in volumetric analysis of multiple sclerosis lesions Journal of computer assisted tomography 1993 17 2 200 10.1097/00004728-199303000-00007 8454745

[B4] Rovaris M Reproducibility of brain MRI lesion volume measurements in multiple sclerosis using a local thresholding technique: effects of formal operator training European neurology 1999 41 4 226 230 10.1159/000008055 10343154

[B5] Wack DS Improved assessment of multiple sclerosis lesion segmentation agreement via detection and outline error estimates BMC Med Imaging 2012 12 1 17 10.1186/1471-2342-12-17 22812697 PMC3428663

[B6] Hamarneh G 3D live-wire-based semi-automatic segmentation of medical images 2005 Proc. SPIE 5747, Medical Imaging 2005: Image Processing, 1597: Citeseer

[B7] Schenk A Prause G Peitgen H *Efficient semiautomatic segmentation of 3d objects in medical images*. in *Medical Image Computing and Computer-Assisted Intervention - MICCAI* Publisher: Springer-Verlag Berlin Heidelberg 2000 1935 186 195

[B8] Derraz F Semi-automatic segmentation of multiple sclerosis lesion based active contours model and variational Dirichlet process CMES 2010 67 2 95 118

[B9] Cuadra M Atlas-based segmentation of pathological MR brain images using a model of lesion growth IEEE Trans Med Imaging 2004 23 10 1301 1314 10.1109/TMI.2004.834618 15493697

[B10] Rovaris M A comparison of conventional and fast spin-echo sequences for the measurement of lesion load in multiple sclerosis using a semi-automated contour technique Neuroradiology 1997 39 3 161 165 10.1007/s002340050384 9106285

[B11] Freifeld O Greenspan H Goldberger J Multiple sclerosis lesion detection using constrained GMM and curve evolution Journal of Biomedical Imaging 2009 2009 1 13 10.1155/2009/715124 PMC2742654 19756161

[B12] Chao M Automated contour mapping with a regional deformable model Int J Radiat Oncol Biol Phys 2008 70 2 599 608 10.1016/j.ijrobp.2007.09.057 18207035

[B13] Lu W Automatic re-contouring in 4D radiotherapy Phys Med Biol 2006 51 1077 10.1088/0031-9155/51/5/002 16481679

[B14] Di Perri C Signal abnormalities on 1.5 and 3 Tesla brain MRI in multiple sclerosis patients and healthy controls: a morphological and spatial quantitative comparison study Neuroimage 2009 47 4 1352 1362 10.1016/j.neuroimage.2009.04.019 19371784

[B15] Jenkinson M Smith S A global optimisation method for robust affine registration of brain images Medical image analysis 2001 5 2 143 156 10.1016/S1361-8415(01)00036-6 11516708

[B16] Jenkinson M Improved optimization for the robust and accurate linear registration and motion correction of brain images Neuroimage 2002 17 2 825 841 10.1006/nimg.2002.1132 12377157

[B17] Zijdenbos A Morphometric analysis of white matter lesions in mr images: methodand validation IEEE Trans Med Imaging 1994 13 4 716 724 10.1109/42.363096 18218550

[B18] Lladó X Segmentation of multiple sclerosis lesions in brain MRI: a review of automated approaches Information Sciences 2011 186 164 185

[B19] Lladó X Automated detection of multiple sclerosis lesions in serial brain MRI Neuroradiology 2011 54 787 807 22179659 10.1007/s00234-011-0992-6

[B20] Udupa JK Multiple sclerosis lesion quantification using fuzzy-connectedness principles Medical Imaging, IEEE Transactions on 1997 16 5 598 609 10.1109/42.640750 9368115

[B21] Sajja B *A unified approach for lesion segmentation on MRI of multiple sclerosis*. in *Engineering in Medicine and Biology Society, 2004. IEMBS'04. 26th Annual International Conference of the IEEE* IEEE 2004 1 1778 1781 10.1109/IEMBS.2004.1403532 17272052

[B22] Alfano B Automated segmentation and measurement of global white matter lesion volume in patients with multiple sclerosis J Magn Reson Imaging 2000 12 6 799 807 10.1002/1522-2586(200012)12:6<799::AID-JMRI2>3.0.CO;2-# 11105017

[B23] Admiraal-Behloul F Fully automatic segmentation of white matter hyperintensities in MR images of the elderly Neuroimage 2005 28 3 607 617 10.1016/j.neuroimage.2005.06.061 16129626

[B24] Anbeek P Probabilistic segmentation of white matter lesions in MR imaging Neuroimage 2004 21 3 1037 1044 10.1016/j.neuroimage.2003.10.012 15006671

[B25] Khayati R Fully automatic segmentation of multiple sclerosis lesions in brain MR FLAIR images using adaptive mixtures method and Markov random field model Computers in biology and medicine 2008 38 3 379 390 10.1016/j.compbiomed.2007.12.005 18262511

[B26] Magnano C Cine cerebrospinal fluid imaging in multiple sclerosis J Magn Reson Imaging 2012 36 4 825 834 10.1002/jmri.23730 22733409

[B27] Datta S Segmentation and quantification of black holes in multiple sclerosis Neuroimage 2006 29 2 467 474 10.1016/j.neuroimage.2005.07.042 16126416 PMC1808226

